# Supplementary material for: Validation of the Quality Analysis of Medical Artificial Intelligence (QAMAI) tool: a new tool to assess the quality of health information provided by AI platforms
Source: Eur Arch Otorhinolaryngol. 2024 May 4;281(11):6123–31. doi: 10.1007/s00405-024-08710-0 (PMC11512889; doi:10.1007/s00405-024-08710-0)
Supplement: Supplementary file 1 — Supplementary table 1. Questions and answers given by ChatGPT-4. Incorrect references are shown in bold (DOCX 53 KB) [file 405_2024_8710_MOESM1_ESM.docx]

| Supplementary table 1. Questions and answers given by ChatGPT-4. | | | | |
| --- | --- | --- | --- | --- |
| Category | N° | Question | Answer | QAMAI score  Median [IQR] |
| Patients’ questions | 1 | I have been diagnosed with squamous cell carcinoma of the tongue, the surgeon told me that it is necessary to have surgery to remove the tumor and the lymph nodes in the neck and to reconstruct the tongue with tissue taken from the forearm. I told the surgeon that I need to think about it but I don't feel like undergoing the operation. What are the potential consequences if I decide not to have surgery? | It's completely normal to have concerns and uncertainties about such a significant surgery. Your decision should be made in conjunction with your healthcare providers, taking into account your personal values, priorities, and lifestyle.  If you choose not to have surgery, there are several potential consequences:   1. Tumor progression: Without intervention, the tumor is likely to continue to grow. This can lead to increased pain, and difficulty eating or speaking, severely impacting your quality of life. [1] 2. Spread of the cancer (metastasis): One of the major risks of untreated cancer is the potential for it to spread to other parts of the body. In the case of oral cancers, the first place they often spread to is the lymph nodes in the neck. From there, they can metastasize to distant organs such as the lungs. [2] 3. Reduced survival rates: The prognosis for oral cancers is generally better the earlier it is treated. Delaying or refusing surgery could potentially reduce survival rates. [3] 4. Complications due to tumor location: Depending on the size and location of the tumor, complications such as airway obstruction or difficulty swallowing can occur. [4]   In terms of what you should do, there are several actions you might consider:  Consult with your healthcare team: It's crucial to have an open dialogue with your healthcare providers. They can provide more detailed information about your specific case and answer any questions you have about the surgery or alternative treatments.  Seek a second opinion: A second opinion can provide additional perspectives on your diagnosis and treatment plan, helping you make a more informed decision.  Consider counseling or mental health support: This can be a very stressful and emotional time. Counseling or mental health support can help you navigate these feelings and provide strategies for coping.  Look into clinical trials or other treatments: There may be clinical trials or other treatments available that could provide alternatives to surgery. You can discuss these options with your doctor.  Learn about palliative care: If you choose not to have curative treatment, you might still consider palliative care. This is care designed to provide comfort and improve quality of life for patients with serious illnesses. [5]  Please take your time to make the best decision for yourself.  References:   1. American Cancer Society. (2020). Oral Cavity and Oropharyngeal Cancer. https://www.cancer.org/cancer/oral-cavity-and-oropharyngeal-cancer.html 2. National Cancer Institute. (2021). Oral Cancer. https://www.cancer.gov/types/head-and-neck/patient/oral-treatment-pdq 3. National Institute of Dental and Craniofacial Research. (2021). Oral Cancer 5-Year Survival Rates. https://www.nidcr.nih.gov/research/data-statistics/oral-cancer/survival-rates 4. **Marur S, Forastiere AA. (2020). Head and Neck Squamous Cell Carcinoma: Update on Epidemiology, Diagnosis, and Treatment. Mayo Clin Proc. https://www.ncbi.nlm.nih.gov/pmc/articles/PMC7082491/** 5. **National Hospice and Palliative Care Organization. (2021). What is Palliative Care? https://www.nhpco.org/patients-and-caregivers/what-is-palliative-care/** | 25 [23.5-28.5] |
| Patients’ questions | 2 | A month ago, I underwent the removal of a basal cell carcinoma from the skin of my cheek. The stitches were removed 20 days ago. The scar is still very noticeable, but the surgeon told me that's normal. In your opinion, is there any problem? What can I do to help the scar heal better? | Post-surgery, scars are typically noticeable and can take months, or even a year or more, to fade and become less noticeable. It is normal to have a noticeable scar 20 days after the removal of the stitches, especially for surgeries on sensitive areas such as the face. The rate of scar healing can be affected by many factors including the size and depth of the wound, your age, and your genetics.  There are several things you can do to help improve the appearance of the scar:   1. Sun Protection: Protect the scar from the sun, as sun exposure can cause the scar to darken and become more noticeable. Apply a broad-spectrum sunscreen of at least SPF 30 before going outside. 2. Scar Massage: Gentle massage of the scar can help to reduce scar thickness and improve its appearance. However, please consult with your healthcare provider before starting scar massage to ensure it's safe and appropriate in your case. 3. Topical Treatments: Silicone sheets and gels have been shown to be effective for reducing scar thickness and discoloration. Vitamin E oil is another option some people find helpful, although scientific evidence of its effectiveness is limited. 4. Healthy Lifestyle: Maintaining a healthy lifestyle that includes a balanced diet and regular exercise can help promote better healing.   Remember to consult with your healthcare provider before starting any new treatment or remedy to ensure it is safe and suitable for your specific situation.  The information I have provided comes from the following sources:   1. Journal of Cutaneous and Aesthetic Surgery, "Postoperative Scar Management" (https://www.ncbi.nlm.nih.gov/pmc/articles/PMC3022978/) 2. **American Academy of Dermatology Association, "How to Minimize the Look of Scars" (https://www.aad.org/public/everyday-care/injured-skin/burns/minimize-scars)**   Again, this information is intended to be general in nature, and specific causes of poor healing or complications should be discussed directly with your healthcare provider. | 24 [23-25] |
| Patients’ questions | 3 | I have to undergo a superficial parotidectomy to remove an adenoma. I am a bit worried. What could be the complications?" | I understand your concerns. A superficial parotidectomy is a surgical procedure to remove part of the parotid gland, which is one of the salivary glands in the face. This procedure is often performed to remove benign (non-cancerous) tumors, like an adenoma, from the gland.  As with any surgery, there are potential risks and complications, including:   1. Facial nerve injury: The parotid gland is closely related to the facial nerve, which controls movements of the face. Injury to the facial nerve is a risk in parotid surgery. This can lead to weakness or paralysis of part of the face, which can be temporary or permanent. 2. Frey syndrome: This is a condition in which sweating occurs on the cheek while eating. It's caused by damage to the nerve that controls sweating in the face during surgery. 3. Salivary fistula or sialocele: This is a condition where saliva leaks from the wound or forms a pocket under the skin. 4. Infections, bleeding, and hematoma formation. 5. Scarring. 6. Numbness in the earlobe or around the incision.   Your surgeon will use techniques to minimize these risks and will monitor you closely after the operation for any signs of complications. It is important to discuss any concerns with your surgeon before the procedure.  Sources for this information include:   1. **The British Association of Otorhinolaryngology - Head & Neck Surgery, "Parotidectomy" (https://www.entuk.org/parotidectomy)** 2. American Academy of Otolaryngology–Head and Neck Surgery, "Salivary Glands" (https://www.entnet.org/content/salivary-glands) 3. **JAMA Network, "Risk Factors for Frey Syndrome After Parotidectomy: A Systematic Review and Meta-analysis" (https://jamanetwork.com/journals/jamaotolaryngology/fullarticle/2664067)**   Again, please discuss these potential complications and any other concerns you have with your surgeon, who will be able to provide the most accurate information based on your specific condition and the surgical approach they plan to use. | 24 [23-24.5] |
| Patients’ questions | 4 | My 17-year-old daughter has an infected cyst in her neck. She underwent an ultrasound that resulted in a "thyroglossal duct cyst." What is this? What are the possible treatments? | A thyroglossal duct cyst is a benign (non-cancerous) condition that can occur in children and adolescents. It happens when a small canal, known as the thyroglossal duct, doesn't close and disappear as it should during embryonic development. This duct is responsible for the descent of the thyroid gland to its final location in the neck during fetal development. If the duct remains open, it can accumulate fluid and form a cyst.  Thyroglossal duct cysts typically appear as a midline neck lump, often near the hyoid bone. These cysts can become more noticeable and tender when infected.  Regarding treatment, the standard approach to a thyroglossal duct cyst is surgical removal. The specific surgical procedure is called a Sistrunk procedure. This procedure not only removes the cyst, but also the tract leading to it and a portion of the bone from which the tract arises, in order to prevent recurrence. Antibiotics may be prescribed if there's an infection, and the surgery may be delayed until the infection is controlled.  Your daughter's healthcare provider will be able to give you more detailed information about her condition and the best treatment options based on her specific case.  The sources I used to gather this information include:   1. **Mayo Clinic, "Thyroglossal duct cyst" (https://www.mayoclinic.org/diseases-conditions/thyroglossal-duct-cyst/symptoms-causes/syc-20377507)** 2. **American Academy of Otolaryngology–Head and Neck Surgery, "Pediatric Thyroglossal Duct Cyst" (https://www.entnet.org/content/pediatric-thyroglossal-duct-cyst)** 3. **Medscape, "Thyroglossal Duct Cyst Surgery" (https://emedicine.medscape.com/article/848401-overview)**   Remember to always consult with your daughter's healthcare provider for personalized advice. | 22 [20.5-22] |
| Patients’ questions | 5 | I am a 53-year-old woman, suffering from hypertension and neck pain for about two years, following a road accident. For about two weeks, I have been feeling a sense of tension in my neck, just below the jaw. The sense of tension becomes actual pain just before meals, associated with modest swelling of the area. What is the most likely cause of my problem? | Given the specific symptom of pain and swelling below the jaw associated with meal times, one possibility to consider is a salivary gland disorder, such as sialadenitis (inflammation of a salivary gland) or salivary stones (also known as sialolithiasis). This condition can cause pain and swelling of the involved salivary gland, particularly around meal times when saliva production increases.  The glands most often affected are the submandibular glands, located just under the jaw, which might align with the location of your symptoms. However, the parotid glands (in front of the ears) and sublingual glands (under the tongue) can also be affected.  Here are some relevant references to support this:   - **British Medical Journal Best Practice guideline on "Sialadenitis": https://bestpractice.bmj.com/topics/en-us/268** - Medscape's article on "Sialolithiasis": https://emedicine.medscape.com/article/882358-overview   Again, this is only a potential explanation based on your symptoms. It's important to consult with a healthcare provider for an accurate diagnosis. Other potential causes could include musculoskeletal issues, dental problems, or even conditions involving the lymph nodes or thyroid gland. If your symptoms continue or worsen, please seek medical attention. | 22 [21-23] |
| Patients’ questions | 6 | I am a 63-year-old woman. I suffer from hypercholesterolemia and am taking simvastatin, hypertension for which I take losartan, and severe osteoporosis, being treated with alendronic acid. My dentist removed a tooth two months ago. Since then, the area of the procedure has not yet healed, I have pain, and my gum is still open. What is the most likely diagnosis? | Given your current medication use and the symptoms you described, one potential explanation could be a condition known as medication-related osteonecrosis of the jaw (MRONJ). This condition is associated with certain medications, including bisphosphonates like alendronic acid, which you're taking for osteoporosis. MRONJ is characterized by non-healing extraction sockets or exposed bone persisting for more than 8 weeks, which seems to match your symptoms.  Here are some relevant references:   - **"Medication-related osteonecrosis of the jaw: 2020 position statement of the Korean Society for Bone and Mineral Research and the Korean Association of Oral and Maxillofacial Surgeons" in the Journal of Bone Metabolism, 2020 Nov; 27(4): 259–267 (https://www.ncbi.nlm.nih.gov/pmc/articles/PMC7686265/)** - American Association of Oral and Maxillofacial Surgeons position paper on Medication-Related Osteonecrosis of the Jaw—2014 Update (https://www.aaoms.org/docs/govt_affairs/advocacy_white_papers/mronj_position_paper.pdf?pdf=MRONJ-Position-Paper)   However, other potential causes for a non-healing oral wound could include infection, poor oral hygiene, or systemic conditions such as diabetes or immune disorders.  Given the potential seriousness of MRONJ, it's crucial to consult with your healthcare provider as soon as possible. They may need to collaborate with your dentist and possibly a specialist such as an oral and maxillofacial surgeon. The chosen treatment approach will depend on the severity of the condition, and can range from medication adjustments to surgical intervention. | 23 [22-24] |
| Patients’ questions | 7 | For about a month I've had a canker sore on my tongue. My doctor prescribed antibiotic therapy, which didn't provide any benefit. For this reason, I was advised to apply a cortisone gel, but the canker sore continues to hurt and doesn't seem to heal. What is the most likely diagnosis and what should I do?. | Canker sores, also known as aphthous ulcers, are usually benign and self-limiting, often resolving within a couple of weeks. However, when they persist beyond that time, as is your case, or recur frequently, it may indicate a more chronic condition such as Complex Aphthosis or Behcet's Disease. It could also be indicative of a systemic condition like nutritional deficiencies, gastrointestinal diseases (like Celiac or Crohn's disease), or immune disorders. Certain medications may also cause mouth ulcers.   - "Aphthous Ulcers" on Cleveland Clinic: https://my.clevelandclinic.org/health/diseases/10945-aphthous-ulcers-canker-sores   Behçet’s disease is a rare disorder that causes blood vessels to become inflamed. In addition to mouth sores, it also causes skin rashes, eye inflammation, and genital sores.   - **"Behçet’s disease" on Mayo Clinic: https://www.mayoclinic.org/diseases-conditions/behcets-disease/symptoms-causes/syc-20351324**   Complex Aphthosis is a condition characterized by recurrent, multiple, or persistent aphthous ulcers, often with a family history of similar issues.   - "**Complex Aphthosis" on DermNet NZ: https://dermnetnz.org/topics/complex-aphthosis/**   When simple measures like over-the-counter gels and mouthwashes don't seem to help, it's important to see a healthcare provider for evaluation. This may include looking for underlying systemic conditions, and possibly referral to a specialist such as a dermatologist or rheumatologist depending on the suspected cause.  Lastly, it's important to note that oral cancers may also present with persistent ulcers. While less likely, especially if you don't have risk factors such as tobacco or alcohol use, it's important to rule this out.   - "Oral cancer" on Mayo Clinic: https://www.mayoclinic.org/diseases-conditions/mouth-cancer/symptoms-causes/syc-20350997   Consult your healthcare provider about your ongoing symptoms. Depending on the cause, you might need different treatments, from topical therapies (like the corticosteroid you've tried), to systemic treatments like colchicine or thalidomide for more severe aphthosis or Behcet's, or addressing any identified underlying conditions. | 18 [17-19] |
| Theoretical questions | 8 | What are the landmarks to find the main trunk of the facial nerve during a parotidectomy procedure? | A parotidectomy is the surgical removal of the parotid gland, which is the major and the largest of the salivary glands. During this procedure, a critical step is identifying the main trunk of the facial nerve to avoid damaging it. This nerve controls most facial expressions and functions, so injury to it can result in significant complications.  Here are the commonly used landmarks for finding the main trunk of the facial nerve during a parotidectomy:   1. The Tragal Pointer: This is a cartilaginous projection in front of the ear canal. It generally points towards the main trunk of the facial nerve, which is typically located about 1 cm deep and slightly medial to this pointer. 2. The Tympanomastoid Suture Line: This is the junction between the tympanic bone and the mastoid bone of the skull. The main trunk of the facial nerve is usually located just inferior to this suture line. 3. The Posterior Belly of the Digastric Muscle: The facial nerve trunk typically runs parallel and deep (medial) to the posterior belly of the digastric muscle. 4. The Styloid Process: The main trunk of the facial nerve is located medial to the styloid process. 5. The Retromandibular Vein: This vein often crosses the facial nerve. Its relationship can be variable but it usually crosses superficially (laterally) over the lower part of the nerve.   Note that while these landmarks can assist in locating the facial nerve, individual anatomical variation and the specifics of the disease process being treated can alter the nerve’s location. Thus, the surgeon must always be prepared for potential variation. It is also important to note that facial nerve monitoring can be a useful adjunct in these surgeries to help prevent nerve damage.   1. "Gray's Anatomy: The Anatomical Basis of Clinical Practice" for detailed anatomical information. 2. "Cummings Otolaryngology: Head and Neck Surgery" for details on procedures like parotidectomy. | 28 [27-29.5] |
| Theoretical questions | 9 | Can you describe in detail the surgical steps to perform a selective neck dissection of levels I-III? | A selective neck dissection (SND) is a procedure designed to remove lymph nodes from one or more regions of the neck (level I to V). This procedure is less extensive than a radical neck dissection and preserves many important neck structures. The procedure you're referring to is focused on levels I-III.  I must clarify that, as an AI, I generate responses based on a mixture of licensed data, data created by human trainers, and publicly available data. However, I can't provide direct bibliographic references as I do not access specific databases or libraries.  However, the steps for a selective neck dissection (SND) of levels I-III typically include:   1. Positioning and Incision: The patient is positioned supine with the neck extended. A standard incision is made along the anterior border of the sternocleidomastoid muscle. 2. Flap Elevation: The skin and subcutaneous tissues are elevated, either in a subplatysmal plane or down to the level of the investing layer of the deep cervical fascia. 3. Identification and Preservation of Important Structures: Important structures such as the spinal accessory nerve, the internal jugular vein, and the sternocleidomastoid muscle are identified and preserved during a selective neck dissection. 4. Dissection of Levels I-III: The lymphatic tissues in these levels are carefully dissected and removed. These include the submental and submandibular nodes (level I), upper jugular nodes (level II), and middle jugular nodes (level III). 5. Closure: Once the appropriate lymph nodes are removed, the incision is closed in layers.   For further reading, these steps are generally outlined in surgical textbooks like:   1. "Operative Otolaryngology: Head and Neck Surgery" by Eugene N. Myers and Carl H. Snyderman. 2. "Cummings Otolaryngology: Head and Neck Surgery"   It's also important to note that the specifics of the surgical steps may vary based on the individual patient's anatomy, the specific goals of the surgery, and the surgeon's preferences and expertise. This is a high-level overview and does not include all the specific considerations that might be needed in a given case. As such, it should not replace consultation with a trained medical professional. | 18 [16-19] |
| Theoretical questions | 10 | What are the absolute indications for the surgical treatment of an orbital floor fracture? | An orbital floor fracture, also known as a "blowout fracture," is a traumatic injury to the bones of the eye socket, specifically the floor of the orbital cavity. The decision to proceed with surgical repair depends on several factors, including the severity of the fracture, the patient's symptoms, and the presence of specific clinical findings.  Absolute indications for the surgical treatment of an orbital floor fracture generally include:   1. Diplopia (double vision) with a positive forced duction test: This is when the patient has double vision and there is physical resistance to the movement of the eye due to entrapment of extraocular muscles. 2. Large orbital floor fractures: Surgery is usually indicated when more than 50% of the orbital floor is involved, particularly if there are symptoms of enophthalmos (sunken eye) or diplopia. 3. Enophthalmos: Surgery may be needed if there is significant sinking of the eye in the socket, typically greater than 2mm compared to the unaffected side. 4. Entrapment of the inferior rectus muscle: This can lead to a gaze limitation or diplopia. 5. Orbital emphysema causing an increase in intraorbital pressure.   As for the bibliographic references, as an AI developed by OpenAI, I generate responses based on a mixture of licensed data, data created by human trainers, and publicly available data. I don't access databases or libraries for specific documents or references. However, these indications are generally found in the medical literature and textbooks. Some potential sources for further reading include:   1. "Ophthalmology" by Myron Yanoff and Jay S. Duker. (Elsevier, various editions) 2. "Kanski's Clinical Ophthalmology: A Systematic Approach" by Brad Bowling. (Elsevier, various editions) 3. "Plastic Surgery" by Peter C. Neligan, Eduardo D. Rodriguez. (Elsevier, various editions)   Please consult these or similar textbooks for more detailed information, and always consider the advice of a healthcare professional when making decisions about surgical procedures. It is also important to note that the decision to proceed with surgery should be individualized, taking into account the patient's specific circumstances, the expertise of the surgeon, and the resources available | 24 [22.5-24.5] |
| Theoretical questions | 11 | In which cases is adjuvant radiotherapy necessary after the surgical removal of a squamous cell carcinoma of the tongue? | Adjuvant radiotherapy may be indicated in the following cases:   1. Positive or close surgical margins: If cancer cells are found at the edge of the removed tissue, there's a risk that some cancer may have been left behind. 2. Extracapsular spread: If the cancer has spread beyond the capsule of a lymph node, this significantly increases the risk of local recurrence and distant metastasis. 3. Multiple involved lymph nodes: If there are multiple lymph nodes with cancer, radiotherapy may be recommended. 4. Perineural or vascular invasion: These situations indicate a higher risk of recurrence and are thus typically treated with adjuvant radiotherapy. 5. Advanced T-stage: T3-T4 tumors or tumors with deep invasion (>4mm) are associated with a higher risk of recurrence. 6. Lymphatic or blood vessel invasion: If the cancer has invaded these structures, it may be more likely to spread.   As for bibliographic references, as an AI, I can't provide direct references as I do not access specific databases or libraries. However, these indications are typically outlined in oncology textbooks and guidelines. Some potential sources for further reading include:   1. "AJCC Cancer Staging Manual" by the American Joint Committee on Cancer. (Springer, various editions) 2. "DeVita, Hellman, and Rosenberg's Cancer: Principles & Practice of Oncology" by Vincent T. DeVita Jr., Theodore S. Lawrence, Steven A. Rosenberg. (Wolters Kluwer Health, various editions) 3. National Comprehensive Cancer Network (NCCN) Guidelines for Head and Neck Cancers.   Please consult these or similar resources for more detailed information, and always consider the advice of a healthcare professional when making decisions about cancer treatments. Decisions about the need for adjuvant therapy are usually made in a multidisciplinary setting, taking into account a wide range of factors including the specifics of the patient's disease, overall health status, and preferences. | 27 [23-28.5] |
| Theoretical questions | 12 | What are the available techniques for bone reconstruction of the mandible after mandibulectomy? | Reconstruction of the mandible following mandibulectomy is a complex process, with the goal of restoring function and appearance. The choice of technique depends on various factors, such as the location and extent of the defect, the patient's overall health and preferences, and the surgeon's expertise. Here are some of the main techniques:   1. Bone Grafts: These can be harvested from various sites, such as the fibula, scapula, or iliac crest. Often, these are free flaps, meaning they are transferred along with their blood supply and require microvascular surgery to connect the blood vessels. 2. Vascularized Free Flaps: These include the fibular free flap, the radial forearm free flap, and the scapular/parascapular free flap. These flaps can provide bone, muscle, and skin as needed. 3. Reconstruction Plates: These are often used temporarily to maintain the shape of the mandible, especially in cases where radiation therapy is planned, which may compromise the success of bone grafts. 4. Alloplastic Materials: These synthetic materials can be used, particularly in smaller defects or in patients who are not good candidates for other forms of reconstruction. 5. Distraction Osteogenesis: This technique uses a device to gradually stretch the existing bone and stimulate new bone growth. This method is more commonly used in pediatric patients.   As for bibliographic references, as an AI, I can't provide direct references as I do not access specific databases or libraries. However, these techniques are commonly described in surgical textbooks and literature. Some potential sources for further reading include:   1. "Plastic Surgery" by Peter C. Neligan, Eduardo D. Rodriguez. (Elsevier, various editions) 2. "Grabb and Smith's Plastic Surgery" by Charles H. Thorne. (Wolters Kluwer Health, various editions) 3. "Head and Neck Cancer: Multimodality Management" by Jacques Bernier. (Springer, various editions)   Please consult these or similar resources for more detailed information, and always consider the advice of a healthcare professional when making decisions about surgical procedures. Each of these techniques has its own risks and benefits, and the best choice will depend on the individual patient's circumstances. | 22 [19-24] |
| Theoretical questions | 13 | What are the peculiar clinical characteristics of adenoid cystic carcinomas of the salivary glands that differentiate them from other types of tumors at these sites? Please provide bibliographic references to your sources | Adenoid cystic carcinoma (ACC) is a relatively rare form of cancer that most commonly arises in the salivary glands, although it can occur in other sites as well. It is characterized by several features that set it apart from other salivary gland tumors:   1. Slow but Persistent Growth: ACCs typically grow slowly but relentlessly, and they can often persist for many years or even decades. 2. Perineural Invasion: A characteristic feature of ACCs is their tendency to invade nerves (perineural invasion), which can lead to symptoms such as pain or numbness. This characteristic can also make these tumors more difficult to completely remove surgically. 3. Distant Metastasis: Despite their slow growth, ACCs have a tendency to metastasize to distant sites, especially the lungs. This can occur even many years after initial treatment. 4. Recurrence: ACCs have a high rate of recurrence, again reflecting their persistence and propensity for perineural invasion. 5. Histology: On a microscopic level, ACCs have a unique appearance, often characterized by cribriform, tubular, and solid patterns.   As for bibliographic references, as an AI developed by OpenAI, I generate responses based on a mixture of licensed data, data created by human trainers, and publicly available data. I don't access databases or libraries for specific documents or references. However, these characteristics are typically described in oncology textbooks and literature. Some potential sources for further reading include:   1. "Head and Neck Pathology" by Lester D.R. Thompson and Bruce M. Wenig. (Elsevier, various editions) 2. "Cancer of the Head and Neck" by Myers E.N. and Suen J.Y. (Saunders, various editions) 3. "Head and Neck Cancer: Multimodality Management" by Jacques Bernier. (Springer, various editions)   Please consult these or similar resources for more detailed information, and always consider the advice of a healthcare professional when making decisions about cancer treatments. It's also important to note that while these are general characteristics of ACC, there can be significant variation between individual cases. | 26 [23.5-27.5] |
| Theoretical questions | 14 | What are the differences between tumor thickness and depth of invasion, and what is their prognostic value in tongue carcinomas? Please provide bibliographic references to your sources. | In tongue carcinomas, both tumor thickness and depth of invasion are key factors used to predict outcomes and plan treatment. However, the definitions of these two terms can differ slightly:   1. Tumor Thickness: This refers to the vertical measurement from the tumor's surface to its deepest point of invasion. It's often measured in millimeters, and it's generally assessed from the surface of the epithelium to the deepest invasive cancer cell. 2. Depth of Invasion (DOI): This also measures the extent of tumor invasion from the basement membrane of the epithelium to the deepest point of tumor invasion.   When considering tongue cancers, both tumor thickness and depth of invasion have been shown to be significant predictors of cervical lymph node metastases, which is a major prognostic factor in oral cancer survival.  Traditionally, tumor thickness has been used in many studies as a predictor for neck metastasis and prognosis. However, the 8th Edition of the American Joint Committee on Cancer (AJCC) Staging Manual introduced depth of invasion as a critical measure for oral cavity cancers, including tongue cancers. In this manual, DOI rather than tumor thickness is incorporated into the T staging of oral cavity cancers due to its slightly superior predictive ability for lymph node metastasis and survival.  For bibliographic references:   1. "AJCC Cancer Staging Manual" by the American Joint Committee on Cancer. (Springer, various editions) 2. "Oral and Oropharyngeal Cancer" by C. S. Farah, D. R. Simanovich, B. A. Bennett. (Elsevier, 2020) 3. "Head and Neck Cancer: A Multidisciplinary Approach" by Harrison LB, Sessions RB, Kies MS. (Wolters Kluwer/Lippincott Williams & Wilkins, various editions)   Please consult these or similar resources for more detailed information, and always consider the advice of a healthcare professional when making decisions about cancer treatments. | 26 [27-30] |
| Theoretical questions | 15 | What is the first-line treatment in an asymptomatic patient with an incidental finding of a 1 cm stone in the hilum of the submandibular gland? | The management of asymptomatic salivary gland stones (sialolithiasis), especially in the submandibular gland, can be complex and must be individualized, considering the stone's size, location, the patient's age, health status, and their preference. For a 1 cm stone in the hilum of the submandibular gland, surgical removal of the gland (submandibulectomy) is often considered, due to the difficulty in completely removing stones of this size and location with less invasive methods and to prevent potential future symptoms and complications.  However, if the patient is truly asymptomatic and the risks of surgery are deemed high, observation may be an option with clear communication to the patient about potential symptoms that would warrant medical review.  Less invasive alternatives, like sialendoscopy and extracorporeal shock wave lithotripsy (ESWL), might be considered, especially if symptoms develop. These methods are less invasive but might be less successful for a 1 cm stone located at the hilum.  Bibliographic references:   1. "Salivary Gland Disorders and Diseases: Diagnosis and Management" by Patel Otolaryngology. (Thieme, 2011) 2. **"Sialendoscopy and Lithotripsy for Treatment of Sialolithiasis" by Capaccio P, et al. Ann Otol Rhinol Laryngol. 2004;113(10):839-843.** 3. **"Diagnosis and management of salivary gland disorders" by Koch M, et al. In: UpToDate, Post, TW (Ed), UpToDate, Waltham, MA.**   Please consult these or similar resources for more detailed information, and always consider the advice of a healthcare professional when making decisions about treatment for salivary gland stones. | 14 [14-16] |
| Theoretical questions | 16 | What is the gold standard for evaluating the local extension of malignant tongue tumors?" | Magnetic Resonance Imaging (MRI) is considered the gold standard for evaluating the local extent of malignant tumors of the tongue. MRI is particularly useful in evaluating the depth of invasion and the involvement of the muscles of the tongue, which are crucial factors in planning the surgical approach. It also provides better soft tissue contrast compared to other imaging techniques, allowing for superior characterization of the lesion and its relationship with surrounding structures.  References:   1. Lam P, Au-Yeung KM, Cheng PW, Wei WI, Yuen AP, Trendell-Smith N, Li JH, Ahuja A. Correlating MRI and histologic tumor thickness in the assessment of oral tongue cancer. AJR Am J Roentgenol. 2004 Mar;182(3):803-8. doi: 10.2214/ajr.182.3.1820803. 2. **Abdel Razek AAK, Mukherji SK. State-of-the-art imaging of head and neck cancer. Radiol Clin North Am. 2020 May;58(3):467-487. doi: 10.1016/j.rcl.2020.01.005.** | 23 [22-24] |
| Theoretical questions | 17 | What are the possible surgical approaches for the treatment of tumors of the maxilla and what are the indications for each of them? | The surgical approach to tumors of the maxilla (upper jaw) often depends on the location and extent of the tumor, as well as the expected functional and aesthetic outcomes. Some commonly used surgical approaches include:   1. Caldwell-Luc approach: This is a classic approach often used for smaller tumors that are confined to the maxillary sinus. It involves an incision in the mouth's upper gingivobuccal fold to directly access the maxillary sinus. 2. Weber-Ferguson approach: This approach involves a midfacial degloving and is used for larger tumors of the maxilla. It provides broad exposure of the maxilla and is often used when a total maxillectomy is planned. 3. Le Fort I osteotomy: This approach is often used for tumors that have spread more broadly across the maxilla. The Le Fort I osteotomy involves a horizontal cut above the teeth and allows the entire maxilla to be moved downwards, improving access to the tumor. 4. Midfacial degloving: This is a less invasive approach used for benign tumors or selected malignant tumors. It involves the use of nasal and oral incisions to separate the facial skin from the underlying bone and provide access to the maxillary sinus. 5. Transoral robotic surgery (TORS): This is a relatively new approach that uses a surgical robot to remove tumors in the maxilla. It is less invasive than traditional methods and may be used for certain types of benign or early-stage malignant tumors.   These approaches are typically combined with a neck dissection if there is involvement of the neck lymph nodes. After the tumor is removed, reconstructive surgery may be needed to restore function and appearance.  References:   1. Shah JP, Gil Z. Current concepts in management of oral cancer–surgery. Oral Oncol. 2009 Nov;45(4-5):394-401. 2. Patel UA, Moore BA, Wax M, et al. Impact of pharyngeal closure technique on fistula after salvage laryngectomy. JAMA Otolaryngol Head Neck Surg. 2013 Aug;139(11):1156-62. 3. Hanna EY, Kupferman M, Assad D. Robotic endoscopic surgery of the skull base: a novel surgical approach. Arch Otolaryngol Head Neck Surg. 2007 Dec;133(12):1209-14. 4. Chaturvedi P, Datta S, Arya S, et al. Oral squamous cell carcinoma arising in background of oral submucous fibrosis: a clinicopathologically distinct disease. Head Neck. 2013 Oct;35(10):1404-9. 5. D'Cruz AK, Vaish R, Kapre N, et al. Elective versus Therapeutic Neck Dissection in Node-Negative Oral Cancer. N Engl J Med. 2015 Aug 6;373(6):521-9. | 18 [16-19] |
| Theoretical questions | 18 | What are the serum markers that have shown some reliability in predicting the risk of occult lymph node metastasis in oral squamous cell carcinomas? | As of my knowledge cutoff in September 2021, there is ongoing research into serum markers that can predict the risk of occult lymph node metastasis in oral squamous cell carcinoma. However, it is important to note that, to date, no single biomarker has been definitively established as reliable and universally accepted for this purpose.  Several biomarkers have shown promise and are being investigated further. These include:   1. **Cytokeratin 19 fragment (CYFRA 21-1):** Elevated levels of CYFRA 21-1 have been associated with nodal metastasis in patients with oral squamous cell carcinoma (OSCC) [Huang, et al., 2016]. 2. **Squamous cell carcinoma (SCC) antigen:** SCC antigen is a serological marker used primarily for SCC of the cervix, lung, and head and neck. Elevated levels have been associated with poor prognosis and higher stage disease in OSCC [Khalfaoui, et al., 2016]. 3. **C-reactive protein (CRP):** Elevated serum levels of CRP have been associated with lymph node metastasis and poor prognosis in OSCC [Chen, et al., 2015].   Again, while these markers have shown some promise, further research is needed to definitively establish their reliability. Additionally, the use of these markers should be considered as a part of a broader diagnostic approach, including imaging and clinical evaluation, rather than as standalone predictors.  References:   1. **Huang, S. H., et al. (2016). Development and validation of a risk stratification model for regionally advanced oropharyngeal cancer. European Journal of Cancer, 52, 1–9.** 2. **Khalfaoui, T., et al. (2016). The clinical relevance of the metabolism of prostate cancer; zinc and tumor suppression: connecting the dots. Molecular Cancer, 15(1), 1-15.** 3. **Chen, H. H., et al. (2015). The prognostic significance of APC gene mutation and miR-21 expression in advanced-stage colorectal cancer. Colorectal Disease, 15(11), 1367-1374.** | 15 [13-17] |
| Theoretical questions | 19 | What conditions are considered precancerous in the oral cavity, and what is the risk of degeneration into cancer? | Oral precancerous conditions are lesions or conditions that have the potential to transform into cancer. These include:   1. Leukoplakia: This is the most common precancerous lesion, appearing as a white patch in the mouth that cannot be rubbed off. It carries a risk of transformation into oral cancer, with a rate of malignant transformation that varies greatly, ranging from less than 1% to about 20% in studies with long-term follow-up. 2. Erythroplakia: This appears as a red patch in the mouth and has a higher risk of cancerous change than leukoplakia. Studies have reported that more than half of erythroplakia cases show dysplasia or carcinoma at the time of biopsy. 3. Oral submucous fibrosis: This is a chronic condition often associated with the chewing of betel nut, especially in Asian populations. It's characterized by inflammation and progressive fibrosis of the submucosal tissues. The malignant transformation rate ranges from 7% to 30% over a 10-year period. 4. Oral lichen planus: This is a chronic inflammatory condition that can affect the skin and mucous membranes, including the mouth. The risk of malignant transformation is estimated to be around 1%. 5. Actinic cheilitis: This is a potentially malignant disorder, mainly affecting the lower lip, caused by chronic sun exposure. It carries a risk of transformation into squamous cell carcinoma, with estimated rates varying from less than 1% to 10%. 6. Oral dysplasia: This is a histopathological diagnosis that indicates abnormal growth of cells within the oral cavity. The risk of progression to cancer varies widely depending on the degree of dysplasia, with the risk being higher for severe dysplasia or carcinoma in situ.   Management of these conditions often involves eliminating potential carcinogens (such as tobacco and alcohol), regular follow-up, and sometimes surgical removal or other treatments for high-risk lesions.  References:   1. Warnakulasuriya S, Johnson NW, van der Waal I. Nomenclature and classification of potentially malignant disorders of the oral mucosa. J Oral Pathol Med. 2007;36(10):575-80. 2. Mehrotra R, Gupta A, Singh M, Ibrahim R. Application of cytology and molecular biology in diagnosing premalignant or malignant oral lesions. Mol Cancer. 2006;5:11. 3. Petti S. Pooled estimate of world leukoplakia prevalence: a systematic review. Oral Oncol. 2003;39(8):770-80. 4. Mello FW, Miguel AFP, Dutra KL, Porporatti AL, Warnakulasuriya S, Guerra ENS, Rivero ERC. Prevalence of oral potentially malignant disorders: a systematic review and meta-analysis. J Oral Pathol Med. 2018;47(7):633-640. | 25 [24-25.5] |
| Theoretical questions | 20 | What tissue components can be harvested in a flap based on the pedicle of the circumflex scapular artery? | The circumflex scapular artery flap, also known as the scapular free flap, is a versatile option for the reconstruction of many different kinds of defects. Depending on the needs of the reconstruction, different tissues can be harvested with the scapular free flap, including:   1. Skin and subcutaneous tissue: This can be used to cover soft tissue defects. 2. Muscle: The scapular flap can include portions of the latissimus dorsi or teres major muscles, which can provide bulk for filling defects or can be used for functional muscle transfers. 3. Bone: The lateral border of the scapula can be included in the flap for bone reconstruction. This could be useful in mandibular reconstructions. 4. Fascia: The fascia of the muscles can also be included in the flap.   The selection of which tissue components to include in the flap will depend on the specific requirements of the reconstruction, the patient's overall health status, and the expertise of the surgeon.  References:   1. **Shindo ML, Costantino PD, Nalbone VP, Rice DH. The scapular/parascapular flap in head and neck reconstruction. Arch Otolaryngol Head Neck Surg. 1993 Mar;119(3):302-8.** 2. **Smith RB, Apostolakis LW, Karnell LH, Koch BB, Robinson RA, Zhen W, Menck HR, Hoffman HT. National Cancer Data Base report on osteosarcoma of the head and neck. Cancer. 2003 Nov 1;98(9):1670-80.** 3. Pellini R, Mercante G, Spriano G. Step-by-step mandibular reconstruction with free fibula flap modelling. Acta Otorhinolaryngol Ital. 2012 Dec;32(6):405-9. | 22 [21-22] |
| Theoretical questions | 21 | What are the therapeutic options for Frey's Syndrome? | Frey's syndrome, also known as gustatory sweating, is a condition where sweating and flushing occur in the area of the cheek adjacent to the ear, often associated with eating certain foods. It is thought to occur due to damaged nerve fibers from the parotid gland that, during regeneration, abnormally reconnect to sweat glands instead of salivary glands. This syndrome often occurs as a sequela of parotid gland surgery but can also result from trauma, infection, or other causes of damage to the parotid gland.  Here are some therapeutic options:   1. Topical antiperspirants: Mild cases of Frey's syndrome may be treated with topical antiperspirants applied to the affected area of the skin. 2. Botox (botulinum toxin) injections: This treatment, administered directly to the affected area, can provide relief from symptoms. It works by blocking nerve signals to the sweat glands, but the effect is usually temporary and repeat injections are required every few months. 3. Neurosurgery: In severe cases, a surgical procedure called neurectomy, which involves cutting the responsible nerve, can be performed. 4. Topical Glycopyrrolate: This is another type of treatment that is applied to the skin to decrease sweating. 5. Surgical Intervention: A barrier may be created between the parotid gland and the skin during initial parotid surgery to prevent the development of Frey's syndrome, but this is more of a preventative measure rather than treatment.   As with all medical interventions, the choice of treatment should be individualized based on the patient's symptoms, overall health, and personal preferences.  References:   1. Dulguerov P, Quinodoz D, Cosendai G, Piletta P, Lehmann W. Prevention of Frey syndrome during parotidectomy. Arch Otolaryngol Head Neck Surg. 1999 Dec;125(12):1335-8. 2. **Drummond M, Frey’s syndrome: treatment with topical glycopyrrolate. The Annals of The Royal College of Surgeons of England. 2004;86(5):327-328.** 3. Laskawi R, Drobik C, Schönebeck C. Up-to-date report of botulinum toxin type A treatment in patients with gustatory sweating (Frey's syndrome). Laryngoscope. 1998 Mar;108(3):381-4. | 23 [21.5-24.5] |
| Clinical scenarios | 22 | I am a head and neck surgeon. I have a patient with squamous cell carcinoma of the skin in the temporal region. On CT scan, there is evidence of a suspected lymph node metastasis at the homolateral level II. What is the recommended procedure for this patient? | In this case, the typical approach to managing squamous cell carcinoma (SCC) of the skin in the temporal region with a clinically suspected level II lymph node metastasis would involve the following steps:   1. Excision of the Primary Tumor: The SCC in the temporal region should be excised with appropriate margins. This will typically involve a wide local excision with the aim of achieving clear margins. 2. Neck Dissection: Given the clinical suspicion of a level II lymph node metastasis, a selective neck dissection of at least levels I, II, and III would typically be recommended. This approach is based on the lymphatic drainage patterns of the scalp and face, which most commonly involve these nodal levels.   However, the specifics of lymph node dissection can vary based on individual patient and tumor characteristics. Some clinicians might choose to also include level IV or perform a parotidectomy in the surgical plan, especially if there are concerns about further lymphatic spread.  For bibliographic references:   1. **"Comprehensive Management of Skin Cancer: Open and Endoscopic Surgery" by Victor Zoccali, et. al. (Springer, 2018)** 2. **"Cutaneous Squamous Cell Carcinoma: A Review of High-Risk and Metastatic Disease" by Que SKT, et. al. American Journal of Clinical Dermatology. 2018;19(4):491-508**. 3. "Head and Neck Cancer: A Multidisciplinary Approach" by Harrison LB, Sessions RB, Kies MS. (Wolters Kluwer/Lippincott Williams & Wilkins, various editions)   Always remember that patient management should be individualized, and decisions should be based on a multidisciplinary discussion involving all members of the care team, and considering the overall health status, preferences, and values of the patient. | 19 [17-20] |
| Clinical scenarios | 23 | I am a head and neck surgeon. I have a patient with squamous cell carcinoma of the mobile tongue, clinically staged as T4a and crossing the midline. There are no evident suspicious lymph nodes. The patient has multiple comorbidities which preclude the use of a free flap. What is the treatment for this type of tumor? What are the reconstructive options? Please provide bibliographic references to your sources | In the case of a T4a squamous cell carcinoma (SCC) of the mobile tongue, management will often involve a multidisciplinary approach. The primary treatment is surgical and typically involves a glossectomy to remove the tumor, with the extent of the glossectomy (partial, hemi-, or total) determined by the size and location of the tumor. The decision to perform a neck dissection even in the absence of evident suspicious lymph nodes should be considered given the propensity of SCC to metastasize to regional lymph nodes.  Given the patient's comorbidities and the inability to perform a free flap, locoregional flap reconstruction might be considered, such as the use of a pedicled myocutaneous flap (for example, pectoralis major myocutaneous flap). The choice of the flap depends on several factors including the size of the defect, the available tissue donors, the patient's overall health status, and the surgeon's expertise.  Postoperative radiation therapy may be considered depending on the final pathological findings, such as positive margins or perineural invasion, and the patient's overall health status.  Please note that this is a complex clinical scenario, and management should be individualized to the patient's needs, health status, and personal preferences.  For bibliographic references:  "Cancer of the Oral Cavity and Pharynx" by Flint PW, Haughey BH, Lund VJ, et al, in Cummings Otolaryngology: Head & Neck Surgery, 6th ed. (Elsevier, 2015)  "Reconstruction of the Tongue and Mouth Floor" by Guo L, et al. in Current Concepts in Head & Neck Surgery and Oncology. (Thieme, 2020)  "Head and Neck Cancer: A Multidisciplinary Approach" by Harrison LB, Sessions RB, Kies MS. (Wolters Kluwer/Lippincott Williams & Wilkins, various editions)  Always consult with a multidisciplinary team and consider the most current clinical guidelines and the patient's preferences and values when deciding on a treatment plan. |  |
| Clinical scenarios | 24 | I am a head and neck surgeon, I have examined a patient with a lesion of the lateral wall of the pharynx. I performed a biopsy that resulted in moderately differentiated squamous cell carcinoma. HPV DNA research was positive. The contrast MRI staged the lesion as T2N0M0. What is the treatment of choice in this case?" | The choice of first-line treatment for a T2N0M0, HPV-positive squamous cell carcinoma of the pharynx involves considering several factors including patient's overall health status, preference, and the exact location and extent of the tumor.  As of the knowledge cut-off in September 2021, the NCCN guidelines (National Comprehensive Cancer Network, version 2.2021 - Head and Neck Cancers) recommend considering either surgery or radiation therapy as the first-line treatment options for T2N0M0 oropharyngeal squamous cell carcinoma. Here are the possible choices:   1. Surgery: A pharyngectomy could be done, with the extent of the surgery depending on the exact location and extent of the tumor. After surgery, adjuvant radiation therapy or chemoradiation might be recommended depending on the final pathology report. 2. Radiation Therapy: Definitive radiation therapy could be used as the first-line treatment, especially for patients who are not good surgical candidates or for those who prefer to avoid surgery.   In both cases, a multidisciplinary team should discuss and decide the best treatment strategy, taking into account the patient's overall health status, the potential benefits and risks of the treatment options, and the patient's preferences.  Again, HPV-positive oropharyngeal cancers generally have a better prognosis than HPV-negative cancers, and there are ongoing clinical trials looking at de-intensification of treatment in these patients to reduce long-term side effects. However, as of the latest knowledge update, no definitive changes in standard treatment based on HPV status have been established.  The evolving nature of medical research mandates that the specific treatment plan should be based on the latest evidence and guidelines from reputable cancer organizations. | 23 [20.5-24] |
| Clinical scenarios | 25 | I'm a head and neck surgeon and I am examining for the first time a patient, a 42 years old male with no other significant medical history. The patient has been experiencing right facial pain, foul nasal discharge and nasal obstruction for 3 months. The computed tomography shows complete opacification of the right maxillary and ethmoid sinus with cortical bone focal erosion over the second upper right molar. The endoscopy shows purulent discharge in the right nasal cavity. What would the head and neck surgeon think about the diagnosis? How would the doctor manage the patient from a clinical and surgical point of view? Provide detailed diagnosis and - if needed - drugs and surgical procedures the head and neck surgeon would employ in this case. | Based on the description you provided, the patient seems to present with signs and symptoms of chronic sinusitis with possible complication of an odontogenic infection. The presence of chronic facial pain, foul nasal discharge, and nasal obstruction, combined with complete opacification of the right maxillary and ethmoid sinuses on CT, are suggestive of chronic sinusitis. The erosion over the second upper right molar suggests a possible odontogenic source of infection, potentially an abscess or periapical infection that has spread to the sinus.  The head and neck surgeon or otolaryngologist might consider the following:  Diagnostic steps:   1. Clinical Evaluation: A thorough history and physical examination, along with findings from the CT scan and nasal endoscopy, could help confirm the diagnosis of chronic sinusitis and possible odontogenic infection. 2. Microbiological Investigation: The otolaryngologist might collect a sample of the purulent discharge during nasal endoscopy for culture to identify the causative organism and guide antibiotic therapy. 3. Dental Consultation: A referral to a dentist or an oral surgeon might be necessary to evaluate the second upper right molar, especially if there's a dental infection contributing to the sinusitis.   Management:   1. Medical Therapy: Initially, medical management might involve systemic antibiotics to cover common pathogens seen in sinusitis (streptococci, Haemophilus influenzae, Moraxella catarrhalis) and possibly anaerobes, given the odontogenic source. A common choice might be amoxicillin-clavulanate. Pain control with analgesics and decongestants to decrease edema and promote sinus drainage could be part of the management plan as well. 2. Surgical Intervention: If there is no improvement with medical management or if complications arise, surgical intervention may be considered. This could involve endoscopic sinus surgery (ESS) to improve sinus drainage. In addition, if an odontogenic source is confirmed, treatment might require dental procedures like root canal therapy or tooth extraction. 3. Follow-up Care: This is important to ensure the resolution of the infection, the absence of complications, and to manage any recurrent or persistent symptoms.   Please note that this information is based on the most recent literature up until the knowledge cutoff in September 2021, and you should refer to the latest guidelines and discuss with your team for the best patient care.  References:   1. Orlandi RR, Kingdom TT, Hwang PH, et al. International Consensus Statement on Allergy and Rhinology: Rhinosinusitis. Int Forum Allergy Rhinol. 2016 Feb;6 Suppl 1:S22-209. doi: 10.1002/alr.21695. 2. Chow AW, Benninger MS, Brook I, et al. IDSA clinical practice guideline for acute bacterial rhinosinusitis in children and adults. Clin Infect Dis. 2012 Apr;54(8):e72-e112. doi: 10.1093/cid/cis370. 3. Little RE, Long CM, Loehrl TA, Poetker DM. Odontogenic sinusitis: a review of the current literature. Laryngoscope Investig Otolaryngol. 2018 Apr 2;3(2):110-114. doi: 10.1002/lio2.145. | 23 [22-24] |
| Clinical scenarios | 26 | I'm a head and neck surgeon examining an MMA fighter who reports noticing the sudden appearance of swelling at the left periorbital area after blowing his nose after a match 7 days ago. The swelling gradually disappeared but for about two days he reports diplopia in upward gaze. What would the head and neck surgeon think about the diagnosis? How would the doctor manage the patient from a clinical and surgical point of view? Provide detailed diagnosis and - if needed - drugs and surgical procedures the head and neck surgeon would employ in this case. | Given the history of recent trauma (MMA match), the sudden periorbital swelling after nose blowing, and new-onset diplopia, the likely diagnosis could be an orbital blowout fracture. The increase in intra-nasal pressure during nose blowing could have forced air into the soft tissues (causing swelling) via an existing fracture, while the diplopia may be due to muscle entrapment or nerve damage caused by the fracture.  The management could involve the following steps:  Diagnostic Steps:   1. Clinical Evaluation: A comprehensive physical examination including an ocular examination to assess eye movements, visual acuity, and pupillary responses. 2. Imaging: A facial CT scan, with particular attention to the orbits, could provide a definitive diagnosis by showing the exact location and extent of the fracture.   Management:   1. Observation: Some small fractures without significant symptoms can be managed conservatively with close observation. The patient should avoid nose blowing, sneezing, and heavy lifting to prevent further orbital emphysema. 2. Medical Therapy: Systemic corticosteroids may be considered to reduce swelling and inflammation, especially in the case of muscle entrapment. Antibiotic prophylaxis can be considered as there may be a risk of infection. 3. Surgical Intervention: If the patient has significant symptoms (like persistent diplopia) or signs of muscle entrapment, surgery may be required. This could be performed by an oculoplastic surgeon, a maxillofacial surgeon, or a head and neck surgeon with experience in orbital surgeries. Surgery usually involves repairing the fracture and releasing any entrapped tissues. 4. Follow-up Care: Regular follow-ups to assess the resolution of symptoms, the success of the treatment and to manage any complications are necessary.   Please note that this information is based on the most recent literature up until the knowledge cutoff in September 2021, and you should refer to the latest guidelines and discuss with your team for the best patient care.  References:   1. Burnstine MA. Clinical recommendations for repair of isolated orbital floor fractures: an evidence-based analysis. Ophthalmology. 2002 Aug;109(8):1207-10; discussion 1210-1; quiz 1212-3. 2. Scawn RL, Lim LH, Whipple KM, Dolmetsch AM, Priel A, Korn BS, Kikkawa DO. Outcomes of Orbital Blowout Fracture Repair Performed Beyond 6 Weeks After Injury. Ophthal Plast Reconstr Surg. 2016 Jul-Aug;32(4):296-301. | 22 [21-23] |
| Clinical scenarios | 27 | I'm a general practitioner and I'm seeing a patient who has had, for about 4 months, a new growth on the left cheek, just in front of the ear, which is asymptomatic and has undergone slight enlargement over time. For about 10 days, he has had difficulty closing the left eye and raising the eyebrow. What could be the diagnosis? How should I manage this patient? | This patient's presentation suggests a possible parotid gland mass (given the location of the swelling) that may be compressing or involving the facial nerve (which passes through the parotid gland), given the new onset of facial nerve dysfunction (inability to close the eye or raise the eyebrow).  There are various types of parotid gland tumors, with most being benign (like pleomorphic adenoma or Warthin's tumor), but malignancies can also occur.  The recommended steps for management could be as follows:  Diagnostic Steps:   1. Clinical Evaluation: A thorough physical examination including evaluation of cranial nerves, especially facial nerve function. 2. Imaging: Ultrasound can be a good first-line investigation, but a CT or MRI may be needed for better characterization of the mass and its relationship with the facial nerve. 3. Biopsy: Fine needle aspiration biopsy (FNAB) could be considered for cytological examination to help distinguish between benign and malignant lesions. This should be performed by a specialist (like a head and neck surgeon or an interventional radiologist) due to the risk of facial nerve injury.   Management:   1. Referral: You should consider referring this patient to a specialist such as a head and neck surgeon or an otolaryngologist for further management, which would typically involve surgical removal of the mass. 2. Surgical Intervention: This is generally the primary treatment for parotid masses. The extent of surgery depends on the type and extent of the mass and whether the facial nerve is involved. The surgery should be performed by a specialist with expertise in parotid surgery due to the risk of facial nerve injury.   Please note that this information is based on the most recent literature up until the knowledge cutoff in September 2021, and you should refer to the latest guidelines and discuss with your team for the best patient care.  References:   1. Schmidt RL, Hall BJ, Wilson AR, Layfield LJ. A systematic review and meta-analysis of the diagnostic accuracy of fine-needle aspiration cytology for parotid gland lesions. Am J Clin Pathol. 2011 Jul;136(1):45-59. 2. Zbären P, Schüpbach J, Nuyens M, Stauffer E. The value of fine-needle aspiration cytology of parotid gland masses. Laryngoscope. 2005 Oct;115(10):1798-805. | 22 [20.5-23] |
| Clinical scenarios | 28 | I'm a head and neck surgeon and I'm seeing a patient who has had, for about 2 months, a lateral cervical mass of about 3 cm. After imaging and needle aspiration, the suspected diagnosis of reactive lymphadenopathy of infectious origin was made. What serum blood markers should be requested? | In evaluating a patient with suspected reactive lymphadenopathy due to an infectious cause, there are several blood tests that could help identify possible etiologies:   1. Complete Blood Count (CBC): To assess for leukocytosis or leukopenia, which can suggest certain infections. Also, atypical lymphocytes could be seen in conditions such as infectious mononucleosis. 2. Monospot test: This can be positive in cases of infectious mononucleosis caused by Epstein-Barr virus (EBV). 3. EBV serology: In cases where mononucleosis is suspected but the monospot test is negative. 4. Cytomegalovirus (CMV) serology: CMV can also cause infectious mononucleosis-like syndromes. 5. HIV serology: HIV can cause generalized lymphadenopathy. 6. Toxoplasma gondii serology: To rule out toxoplasmosis which can cause lymphadenopathy. 7. Serologic tests for bacterial infections: Including testing for Bartonella henselae for suspected cat scratch disease, or serologic tests for syphilis or tuberculosis in appropriate clinical situations. 8. Hepatitis B and C serology: Hepatitis can sometimes present with lymphadenopathy.   In the event these tests are negative or inconclusive, or if there are other concerning features (e.g., persistent, growing, or hard lymph nodes, systemic symptoms), an excisional biopsy may be necessary for definitive diagnosis.  Please note that this information is based on the most recent literature up until the knowledge cutoff in September 2021, and you should refer to the latest guidelines and discuss with your team for the best patient care.  References:   1. **Agha Z, Stinson D, Hessel AC, Kandil E, Nuss DW, Walvekar RR. Clinical predictors of malignant and premalignant cervical lymphadenopathy: A systematic review. Otolaryngol Head Neck Surg. 2016 May;154(5):793-802.** 2. **Meyer AK, Lippert BM, Gottschlich S, Pabst R. Staging and therapy of oropharyngeal cancer: Importance of different clinical parameters on the incidence of distant metastases and survival rate. Laryngoscope. 1996;106(9 Pt 1):1196-1201.** 3. **Tan LG, Tan YY, Tan IJ, Tao M, Chow PK. The pattern and predictors of mortality of HIV/AIDS patients with and without hepatitis C co-infection: a dynamic cohort study in Asia. J Viral Hepat. 2015 Dec;22(12):1035-44.** | 23 [22-24.5] |
| Clinical scenarios | 29 | I'm a head and neck surgeon and I've seen a patient with moderately differentiated squamous cell carcinoma of the right retromolar trigone. The patient has a history of bilateral tibia and fibula fracture, which occurred 10 years ago, and severe venous insufficiency of the lower limbs. The patient has no other comorbidities. The CT scan shows infiltration of the underlying bone and a 4x5 cm lesion centered on the retromolar trigone, extending laterally to the cheek and medially to the soft palate, surpassing the midline. The CT also detected 3 rounded lymph nodes < 4 cm, compatible with secondary nodes, at level IIa and III on the right. No distant metastases were detected. What is the staging of the tumor? What treatment is indicated for the tumor? What is the first-line reconstructive technique for this specific case? | The case you have presented is of a patient with moderately differentiated squamous cell carcinoma of the right retromolar trigone with lymph node metastasis and bone involvement.  According to the AJCC Cancer Staging Manual (8th edition), the TNM staging would be T4a (tumor invades through the cortical bone, into deep/extrinsic muscle of tongue, maxillary sinus, or skin of face), N2b (metastasis in multiple ipsilateral nodes, none >6 cm), M0 (no distant metastasis). Hence, the overall stage would be Stage IVA.  The typical first-line treatment for Stage IVA oral cancer is surgery, likely involving a composite resection, including a segmental mandibulectomy due to bone invasion, and a neck dissection on the affected side (at least a selective neck dissection of levels IIa and III). This is typically followed by post-operative radiation therapy, possibly with concurrent chemotherapy, depending on the final pathological findings such as positive margins or extracapsular spread in the lymph nodes.  The patient's severe venous insufficiency in the lower extremities does present a challenge when considering reconstruction, particularly if a fibular free flap was initially considered, which is often a first choice for reconstruction in such cases due to its length, bone quality for implant placement, and inclusion of a skin or muscle paddle for additional soft tissue. In this case, the patient's severe venous insufficiency could increase the risk of complications related to the donor site.  Alternative free flaps that might be considered include the scapula free flap or the radial forearm free flap. Each of these flaps have their own advantages and disadvantages. The scapula free flap can provide a good amount of bone for mandibular reconstruction but does not have the long pedicle that the fibula does, while the radial forearm free flap is a reliable flap but does not provide bone for reconstruction.  In such complex cases, it is important to have an individualized approach and multidisciplinary discussion, involving vascular surgeons if needed, and considering the patient's overall health status, the extent of the defect, and the surgeon's expertise.  References:   1. Amin MB, Edge S, Greene F, et al. (eds). AJCC Cancer Staging Manual. 8th Ed. Springer; 2017. 2. Marur S, Forastiere AA. Head and Neck Squamous Cell Carcinoma: Update on Epidemiology, Diagnosis, and Treatment. Mayo Clin Proc. 2016 Mar;91(3):386-96. 3. Bianchi B, Ferri A, Ferrari S, Copelli C, Sesenna E. Free flaps: outcomes and complications in head and neck reconstructions. J Craniomaxillofac Surg. 2009 Oct;37(7):403-7. 4. Suh JD, Sercarz JA, Abemayor E, Calcaterra TC, Rawnsley JD, Alam D, Blackwell KE. Analysis of outcome and complications in 400 cases of microvascular head and neck reconstruction. Arch Otolaryngol Head Neck Surg. 2004 Aug;130(8):962-6. | 23 [22-24] |
| Clinical scenarios | 30 | A patient underwent a left lateral neck dissection for tongue cancer. Four days after the operation, the drain collected 500 mL of clear, serous fluid in 24 hours. What could be the diagnosis? What are the possible treatments? | The described condition is likely a chyle leak, also known as chylous fistula or chylous leakage, which is characterized by the drainage of a milky-colored fluid from the lymphatic system, often as a complication of neck dissections. The presence of a chyle leak postoperatively is suggested by a high output of fluid from the surgical drain, especially if the fluid appears milky or has a high triglyceride content.  Possible treatments for a chyle leak include:   1. Conservative measures: The first-line treatment typically involves dietary modifications, such as a low-fat diet with medium-chain triglycerides, which are more easily absorbed directly into the bloodstream and bypass the lymphatic system. The patient could also be placed on complete bowel rest with total parenteral nutrition (TPN). Additionally, proper wound care, compression dressings, and elevation of the head can minimize the collection of fluid. 2. Medical management: Octreotide, a somatostatin analogue, may be used to decrease lymph flow and has been reported to help in some cases. 3. Surgical management: If conservative management fails or if the chyle leak is high output, surgical exploration and ligation may be necessary. In some cases, lymphatic-venous anastomosis or thoracic duct embolization may be performed.   It's crucial to monitor the patient for signs of dehydration, malnutrition, and immunodeficiency due to loss of lymphatic fluid. Early recognition and treatment can help to avoid these complications.  References:   1. Roh JL, Yoon YH, Park CI. Chyle leakage in patients undergoing thyroidectomy plus central neck dissection for differentiated papillary thyroid carcinoma. Ann Surg Oncol. 2008 Sep;15(9):2576-80. 2. Campisi CC, Boccardo F, Piazza C, Campisi C. Evolution of chylous fistula management after neck dissection. Curr Opin Otolaryngol Head Neck Surg. 2013 Oct;21(2):150-6. | 24 [22.5-24] |
